# Calsyntenin-1 Negatively Regulates ICAM5 Accumulation in Postsynaptic Membrane and Influences Dendritic Spine Maturation in a Mouse Model of Fragile X Syndrome

**DOI:** 10.3389/fnins.2019.01098

**Published:** 2019-10-18

**Authors:** Ke Cheng, Yu-shan Chen, Chao-xiong Yue, Si-ming Zhang, Ya-Ping Pei, Gui-rong Cheng, Dan Liu, Lang Xu, Hong-xin Dong, Yan Zeng

**Affiliations:** ^1^Brain and Cognition Research Institute, Wuhan University of Science and Technology, Wuhan, China; ^2^Big Data Science and Engineering Research Institute, Wuhan University of Science and Technology, Wuhan, China; ^3^Department of Psychiatry and Behavioral Sciences, Northwestern University, Chicago, IL, United States

**Keywords:** calsyntenin-1 (CLSTN1), intercellular adhesion molecule-5 (ICAM5), dendritic spine, intracellular transport, fragile X syndrome

## Abstract

Fragile X syndrome (FXS) is a neurodevelopmental disorder that causes intellectual disability, as well as the leading monogenic cause of autism spectrum disorders (ASD), in which neurons show aberrant dendritic spine structure. The reduction/absence of the functional FMRP protein, coded by the X-linked Fmr1 gene in humans, is responsible for the syndrome. Targets of FMRP, CLSTN1, and ICAM5, play critical roles in the maturation of dendritic spines, synapse formation and synaptic plasticity. However, the implication of CLSTN1 and ICAM5 in dendritic spine abnormalities and the underlying neuropathologic processes in FXS remain uninvestigated. In this study, we demonstrated that CLSTN1 co-localizes and co-transports with ICAM5 in cultured cortical neurons. Also we showed that shRNA-mediated downregulation of CLSTN1 in cultured WT neurons increases ICAM5 on the surface of synaptic membrane, subsequently affecting the maturation of dendritic spines. Whereas, normalization of CLSTN1 level in *Fmr1* KO neurons reduces ICAM5 abundance and rescues impaired dendritic spine phenotypes. Most importantly, CLSTN1 protein is reduced in the postnatal medial prefrontal cortex of *Fmr1* KO mice, which is correlated with increased ICAM5 levels on the surface of synapses and excessive filopodia-like spines. In conclusion, this study demonstrates that CLSTN1 plays a critical role in dendritic spine formation and maturation in FXS by regulating ICAM5 redistribution.

## Introduction

Dendritic spines are small membranous protrusions that constitute the primary postsynaptic sites of excitatory neurotransmission in the brain. Morphological maturation of spines is a delicate process that starts from the formation of the dendritic filopodium, and followed by the precursors of spines during synapse development (Hotulainen and Hoogenraad, 2010; Korobova and Svitkina, 2010), and is associated with the stabilization and strengthening of synapses (Bradke et al., 2000; Jan and Jan, 2001; Scott and Luo, 2001; Peng et al., 2009). Dysmorphic dendritic arbors correlate to the abnormal development of synaptic connectivity and are being observed in many neurodevelopmental disorders including FXS (Hotulainen and Hoogenraad, 2010; Penzes et al., 2011). FXS is a neurodevelopmental disorder that causes intellectual disability and behavioral deficits, it is also a leading genetic cause of autism spectrum disorder (ASD), in which the neurons show immature postsynaptic dendritic spine protrusions as thin, actin-rich filopodia on the surface of dendrites (Comery et al., 1997; Cruz-Martin et al., 2010; Wijetunge et al., 2014). Previously, it has been demonstrated FXS patients or mice exhibited an overabundance of dendritic spines with immature morphologies in the cortical pyramidal neurons, cortex and hippocampus (McKinney et al., 2005; Grossman et al., 2010; Arroyo et al., 2018), while, intriguingly, reversing dendritic deficits can rescue FXS-related phenotypes (inappropriate social behavior, behavioral inflexibility, altered dendritic spine morphology, and macroorchidism) (Bhattacharya et al., 2016). However, the mechanisms underlying dendritic spine abnormalities in FXS are still not well-understood.

FXS results from the loss of function of fragile X mental retardation protein (FMRP), which is an RNA-binding protein (Penagarikano et al., 2007; Korb et al., 2017). A number of FMRP target mRNAs were previously identified by high-throughput sequencing of RNA isolated by crosslinking immunoprecipitation (HITS-CLIP) (Darnell et al., 2011). The neuron-specific intercellular adhesion molecule 5 (ICAM5), a member of the ICAM family of adhesion proteins (Gahmberg et al., 2014), was identified as one of the FMRP targets (Darnell et al., 2011), but subsequent biological analyses of molecular pathways linked between FXS and FMRP targets are lacking. ICAM5 was firstly identified as a dendrite-specific cell adhesion molecule (Yoshihara and Mori, 1994; Gahmberg et al., 2014). It expresses at the soma-dendritic membrane of the subsets of telencephalic neurons, but not the axonal membrane (Benson and Tanaka, 1998; Mitsui et al., 2005; Gahmberg et al., 2014). Enrichment of ICAM5 protein has been found to slow the maturation of dendritic spines (Yoshihara and Mori, 1994; Mizuno et al., 1997; Matsuno et al., 2006; Tian L. et al., 2007). Ablation of its expression increases functional dendritic maturation, synapse formation (Gahmberg et al., 2014; Kelly et al., 2014), and synaptic contact formation (Ning et al., 2013). Recent studies have characterized the matrix metalloprotein-2 (MMP-2)/MMP-9-induced cleavage of ICAM5 after stimulation of glutamate receptors (Tian X. Y. et al., 2007; Ning et al., 2013) and demonstrated that surface removal of ICAM5 affects filopodia-to-spine transition (Raemaekers et al., 2012). However, the mechanisms of the dynamic changes of ICAM5 and its link with FXS have not been characterized before.

Synaptic adhesion molecule calsyntenin-1 (CLSTN1), a type I transmembrane protein and predominantly expressed in the postsynaptic membranes of pyramidal neurons (Vogt et al., 2001), is another FMRP target (Darnell et al., 2011). It has been shown that CLSTN1 is involved in synapse formation as well as synaptic plasticity (Ikeda et al., 2008; Hoerndli et al., 2009). Several recent studies show that CLSTN1 is important for dendritic spine formation by mediating the fast transport of membrane-bound organelles through interactions with kinesin light chain-1 (Vagnoni et al., 2012; Ster et al., 2014; Um et al., 2014). For example, CLSTN1 regulates the transport of Rab5-containing endosomes and axon branching during sensory neuron development (Ohno et al., 2014). Moreover, it also regulates the trafficking of N-methyl-D-aspartic acid (NMDA) receptors to synapses and promotes synapse maturation (Ster et al., 2014). These studies demonstrate the critical role of CLSTN1 in spine development. However, it remains unclear how the transport function of CLSTN1 impacts key molecule, such as ICAM5 that is known to influence dendritic spine maturation, and how CLSTN1 is involved in neurodevelopmental disorders.

In this study, we investigated the mechanistic link between CLSTN1 and ICAM5 in the regulation of dendritic spine maturation and their roles in spine abnormality in a mouse model of FXS. We found the functional relationship between CLSTN1 and ICAM5 by detecting the co-localization and co-transportation of CLSTN1 and ICAM5 in primary cortical neurons. Moreover, we also demonstrated that CLSTN1 decreases ICAM5 abundance on the membrane surface and rescues impaired dendritic spine phenotypes *in vitro*. Furthermore, we showed that CLSTN1 protein is reduced in adolescent *Fmr1* KO brains, and this reduction is correlated with increased ICAM5 expression and dendritic malformation in the medial prefrontal cortex during a critical postnatal period in *Fmr1* KO mice. Taken together, our results suggest a key role for CLSTN1 in mediating ICAM5 redistribution on postsynaptic membranes, which is necessary for the maturation of dendritic spines during early development. Dysfunction of CLSTN1 may contribute to the onset and progression of FXS.

## Materials and Methods

### Mouse Models and Animal Procedure

*Fmr1* knock out (*Fmr1* KO) (FVB.129P2-Pde6b+ Tyrc-ch Fmr1tm1Cgr/J, RRID: IMSR-JAX: 004624) and wild-type (WT) control (FVB.129P2-Pde6b+ Tyrc-ch/AntJ, RRID: IMSR-JAX: 004828) mice were originally purchased from Jackson Laboratories (Bar Harbor, Maine, USA). The mice were bred in Wuhan University of Science and Technology (Wuhan, China). Animals were kept on a 12:12 h dark-light cycle at 22°C with food and water available *ad libitum*. Each cage (CMJ1 experimental mouse cage, Size: 320 × 230 × 169 mm) housed 4 *Fmr1* KO or WT littermates. Since FXS is mostly prevalent in males (14), only healthy male mice were used in this study. 197 *Fmr1* KO and WT mice were used in the experiment and 5 mice with poor wellbeing (unhealthy or diseased mice) were excluded. For *in vivo* experiments, littermate mice at postnatal 1 (P1), P3, P7, P14, P21, or P30 were first allocated into 4 groups (3 mice/group) by weight, from 1 group one mouse was assigned to Golgi and two mice was used for western blot. In order to minimize animal pain and suffering, the animals were placed in a small animal anesthesia machine (RWD, China, RRID: R510-K1) containing 1–4% isoflurane (RWD, China, RRID: R510-22) for 1–3 min before sacrificed by decapitation. Whole brains were taken out for subsequent experimental procedures. The study was approved by the Wuhan University of Science & Technology ethics committee with the number IACUC-2017032. All procedures and husbandry were in accordance with the National Institutes of Health (NIH) Guide for the Care and Use of Laboratory Animals. A timeline of the *in vivo* experimental procedure is shown in Table 1.

**Table 1:** *In vivo* experimental design.

**Experiment model**	**Experiment**	**P1**	**P3**	**P7**	**P14**	**P21**	**P30**
WT mice	Western blot	*n* = 8	*n* = 8	*n* = 8	*n* = 8	*n* = 8	*n* = 8
	Golgi staining	*n* = 4	*n* = 4	*n* = 4	*n* = 4	*n* = 4	*n* = 4
Fmr1 KO mice	Western blot	*n* = 8	*n* = 8	*n* = 8	*n* = 8	*n* = 8	*n* = 8
	Golgi staining	*n* = 4	*n* = 4	*n* = 4	*n* = 4	*n* = 4	*n* = 4

*P, postnatal*.

### Golgi Impregnation Procedure and Spine Analysis

The method for Golgi staining was adapted from Dong et al. (2012), Tian et al. (2015), and Gao et al. (2016). Briefly, the FD Rapid Golgi Stain Kit (FD Neurotechnologies, Columbia MD, USA) was used according to the manufacturer's standard protocol. The impregnation solution was prepared by mixing equal volumes of Solutions A and B 24 h prior to use and then stored in the dark. Mice brains were quickly and carefully removed from the skull, rinsed in 0.1 M Phosphate Buffer (pH 7.4), immersed in a mixed impregnation solution for 14 days at room temperature in the dark, and then transferred into a C solution for 1 week at 4°C in the dark. Solution C was replaced after 12 h of brain immersion. The brains were washed and coronally sectioned 100 μm using a vibratome (NVSLM1 motorized advance Vibroslice, World Precision Instruments, USA). Brain sections were incubated with Solution D and Solution E at room temperature for 10 min and mounted on adhesive microscope slides. Brain sections were then rinsed, dehydrated, cleared of xylenes, and air-dried at room temperature in the dark. A cover slip was placed on the slide to hold sections in place for visualization in confocal microscopy, and nail hardeners were used to seal the slides to avoid drying. Bright-field images of pyramidal neurons in the medial prefrontal cortex were collected using an Olympus BX51WI Microscope. Dendritic spines were counted using Neurolucida software (version 9.0; Vermont, USA). For morphology characterization of spines, the following categories were used: (1) branched spines with more than one head; (2) thin, filopodia-like protrusions; (3) stubby, short spines without a well-defined spine neck; and (4) mushroom spines with a large bulbous head (Bian et al., 2015). For spine length, we measured protrusions that extended between 0.5 and 5 μm from the parent dendrite protrusions. For spine density measurements (number of spines per 10 μm length of dendrite), we counted all spines that were basal dendritic segments of the secondary dendrites of pyramidal neurons in the cortical layers II/III using Golgi staining images. Only spines that emerged perpendicular to the dendritic shaft were counted. One dendritic branch per neuron, 6 neurons per slice, 8 slices per mouse, and 4 mice per group were used for Golgi analysis. Collectively, this corresponded to 192 dendritic branches for each group of mice. All measurements were done by individuals who were blinded to the genotype of the mice being analyzed.

### DNA Constructs

To construct a shRNA vector to knockdown CLSTN1, we used a lentiviral vector GV118 (Shanghai Genechem Co, Ltd.) and determined that the best target sequence is 5′-TAGTGAAGATAAGCGTCAA-3′ (Figure S1 and Table S1). The scrambled control sequence for shRNA (5′-TTCTCCGAACGTGTCACGT-3′) (control shRNA) was also expressed from the GV118 vector and did not target any known mouse cDNA sequence. The full length of CLSTN1 was amplified using a forward primer (5′-AGGTCGACTCTAGAGGATCCCGCCACCATGCTGCGCCGCCCTGCGCCCGCGCTG-3′) and a reverse primer (5- TCCTTGTAGTCCATACCGTAGCTGAGTGTGGAGTCATCCCATTCCAGCTGTC-3′). Amplified CLSTN1 cDNA was ligated into the GV492 vector (Shanghai Genechem Co., Ltd.) to obtain the EGFP-CLSTN1-lentiviral vector. The full length of ICAM5 was amplified using a forward primer (5′-GAGGATCCCCGGGTACCGGTCGCCACCATGCCGGGGCCTTCGCCAGGGCTGC-3′) and a reverse primer (5′-CACACATTCCACAGGCTAGTCAGGAAGATGTCAGCTGGATAGC-3′). Amplified ICAM5 cDNA was ligated into the GV326 vector (Shanghai Genechem Co., Ltd.) to obtain the Cherry-ICAM5-lentiviral vector.

### Quantitative Real-Time (RT) PCR

We evaluated the mRNA level of CLSTN1 and ICAM5 in Fmr1 KO and WT mice by quantitative RT-PCR. Total RNA was extracted by TRIzol Reagent (Invitrogen, USA) and subsequently synthesized into single-strand cDNA using Superscript II reverse transcriptase (Invitrogen, USA). The cDNA amplification was performed using SYBR Premix Ex Tap (Tli RNaseH Plus, Takara) on the BIO-RAD CFX96 system. Forward/reverse primers used for qRT-PCR are shown in Table S2.

### Cell Culture and Transfection

**Figure 1:**
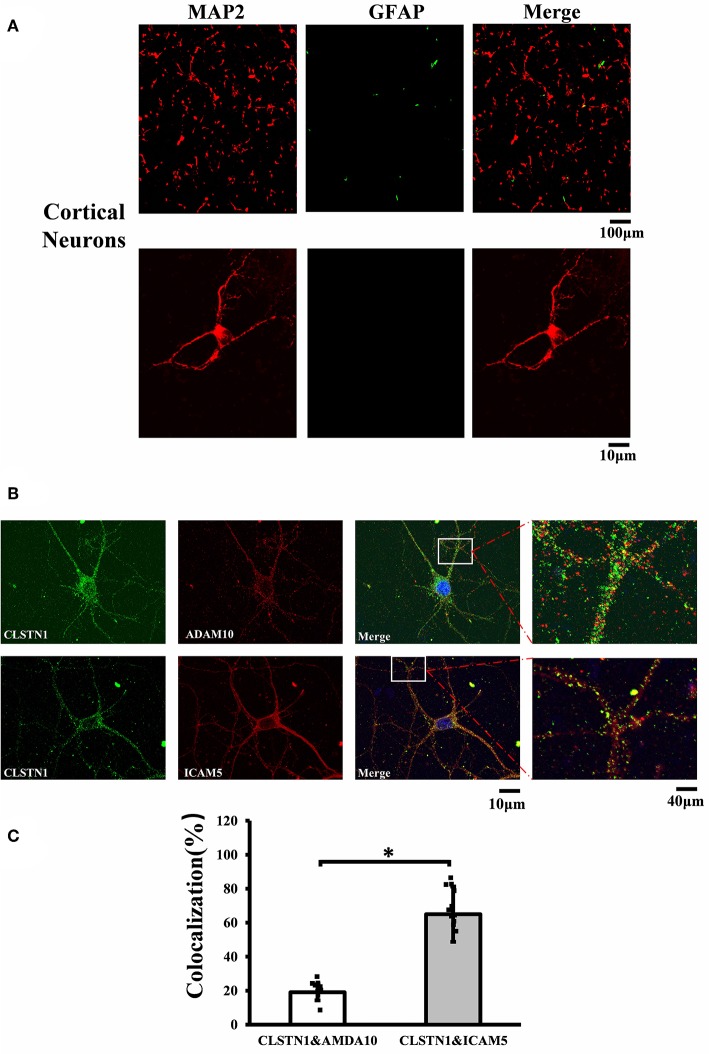
CLSTN1 co-localizes with ICAM5 in mouse cortical neurons. **(A)** Staining for MAP2 and GFAP in cultured cortical neurons. Cultured cortical neurons from the medial prefrontal cortex were fixed and immunostained with MAP2 (red) and GFAP (green) at 14 DIV. **(B)** Co-localization analysis of CLSTN1 and Icam5 or CLSTN1 and ADAM10 is detected in dendrites (*n* = 12). Scale bars = 10 μm. **(C)** Quantification of co-localization was performed in dendrites using the Image J software as described in the methods. Histograms represent the relative intensity of CLSTN1 and ICAM5 or ADAM10. Data are shown as mean ± SD. *p < 0.05 compared to CLSTN1 and ADAM10.

N2a neuroblastoma (N2A) cell line was cultured in Dulbecco's modified Eagle's medium (Gibco, USA) that contained 10% fetal bovine serum (FBS, Gibco, USA), penicillin G (70 mg/l), streptomycin (100 mg/l). Cells were maintained at 37°C in a cell incubator with 5% CO_2_. Primary mouse neurons from the medial prefrontal cortex of WT and *Fmr1* KO neonatal mice were cultured on poly-L-lysine coated glass cover slips (Sigma-Aldrich, RRID: P4707). Cells were grown in neurobasal medium (Gibco, USA, RRID: 10888022) supplemented with 25 μM L-glutamate (abcam, USA, RRID: AB120049), 0.5 mM L-glutamine (Gibco, USA, RRID: 25030-081), 2% B27 (Gibco, USA, RRID: 17504044), and antibiotics (penicillin/streptomycin). Cultures were maintained at 37°C with 5% CO_2_. Half of the media was changed every 3 days. Neurons that infected with 1 × 10^8^ TU/ml (MOI = 10) of lentiviral vectors (EGFP-CLSTN1 and Cherry-ICAM5) were used in co-transport experiment, and CLSTN1 shRNA was used in the CLSTN1 silencing experiment. Infected cells continued to grow in conditioned medium until the neurons were harvested for immunocytochemistry or live cell imaging. To identify cultured neurons, we conducted double labeling of cultures with microtubule associated protein 2 (MAP2, red) and glial fibrillary acidic protein (GFAP, green). Figure 1A shows that the majority of cells are neurons as they were labeled by MAP2 (red) (Figure 1A). A time-line of the *in vitro* experimental procedure is shown in Table 2. All analyses were conducted by individuals who were blinded to the genotype of the mice.

**Table 2:** *In vitro* experimental design.

**Experimental model**	**Experiments**	**DIVl**	**DIV3**	**DIV11**	**DIV14**	**DIV18**
WT neurons	Immunocytochemistry (*n* = 12)	Seeded neurons			lmmunocytochemistry (MAP2 GFAP)	
	Living cell imaging (*n* = 12)		Transfected LV-EGFP-CLSTN1 LV-ICAM5-mCherry	Time lapse (ICAM5 CLSTN1)		
	Western blotting (*n* = 12)		Transfected LV-Cl.STN1-shRNA		Western blotting (CLSTN1 ICAM5 Psd95)	
	Immunocytochemistry (*n* = 12)				lmmunocytochemistry (ICAM5 Psd95)	
	Dil-stained (*n* = 12)					Dil-stained (dendritic spines)
Fmrl KO neurons	Immunocytochemistry (*n* = 12)		Transfected LV-EGFP-CLSTN1			Immunocytochemistry (ICAM5 CLSTN1)
	Western blotting (n = 12)				Western blotting (CLSTN1 ICAM5 Psd95)	
	Dil-stained (*n* = 12)					Dil-stained (dendritic spines)
N2A	Immunocytochemistry (*n* = 8)		Immunocytochemistry (ICAM5 Cl.STN1)			

*DIV, days in vitro*.

### Dil Labeling and Morphometrical Analysis

Dendritic spines were identified using the well-characterized fluorescent marker Dil (1,1′-dioctadecyl-3,3,3′,3′-tetramethylindocarbocyanine perchlorate), as described by Cheng et al. (39). In brief, the neurons on days *in vitro* (DIV) 18 were fixed with 4% paraformaldehyde for 15 min and washed thrice with phosphate buffer saline (PBS). After immersed for 10 min in 2.5 μM Dil (Invitrogen, RRID: D307), cells were washed for three times and examined using a FV1000 confocal-IX81 microscope (FluoView1000-IX81, Olympus, Tokyo, Japan). Image J was used to measure the spine head width and dendritic protrusion length within 100 μm of the cell body. The dendritic spine head area was quantified by manual tracing in the defined region of interest along the dendrites. At least 8 different culturing sections were analyzed for each time point. Spine morphology was estimated according to previously published parameters (Bian et al., 2015).

### Live Neuron Imaging

Cultured neurons were co-infected with EGFP-CLSTN1 and ICAM5-mCherry lentivirus on DIV 3 and time-lapse imaged using Olympus FV1000-IX81 microscope (FluoView1000-IX81, Olympus, Tokyo, Japan) 8 days after co-infection with the following parameters: 100× (1.4 NA), zoom 4, and 512 × 512 frame size; 12 bit depth; and Z-stack of 7 slices with a 0.43 mm interval. Images of EGFP-CLSTN1- and ICAM5-mCherry- positive neurons were captured every second for 5 min per cell under confocal microscopy.

EGFP-CLSTN1- and ICAM5-mCherry- particle mobilities were measured by quantifying the lines in the kymographs. Kymographs were generated with the Multi Kymograph plug-in of the ImageJ software according to the instructions. Each line represents one vesicle. Vertical lines represent stationary vesicles. Oblique lines or curves to the left represent retrograde movements, and lines to the right indicate anterograde transports.

### Immunocytochemistry

Primary cultured neurons on DIV 18 were fixed with 4% paraformaldehyde, extensively washed in 0.1 M PBS, and then permeabilized with 0.1% Triton X-100. After being blocked in 5% bovine serum albumin (BSA) for 1 h at room temperature, neurons were incubated with primary antibodies overnight. The neurons were washed three times and incubated with Alexa Fluor-conjugated secondary antibodies and 4′-6-Diamidino-2-phenylindole (DAPI) as a nuclear counterstain. Images were acquired using a FV1000 confocal microscope (FluoView1000-IX81, Olympus, Tokyo, Japan). For co-localization analysis, at least 4 areas of interest in each coverslip were analyzed. Immunopositive ICAM5, or CLSTN1 and PSD95 puncta were thresholded to visually recognizable punctate labeling. The thresholded puncta in one channel were used to create a mask and then overlaid on the second channel to obtain the overlapping/co-located regions. At least 8 complete neuronal profiles in each of 4 different culture sections were analyzed. Primary antibodies used are as follows: Rabbit MAP2 (Cell Signaling Technology, RRID: 4542S, 1:200); Mouse GFAP (Cell Signaling Technology, RRID: #3670, 1:300); Rabbit CLSTN1 (Santa, RRID: sc133315, 1:200); Goat ICAM5 (Santa, RRID: sc-22028, 1:200); and Mouse PSD95 (Cell Signaling Technology, RRID: 2507S, 1:200). Secondary antibodies used are as follows: Rabbit Cy3 (Jackson, RRID: 305-165-003, 1:400); Mouse Alexa Fluor 488 (Bioss, RRID: bs-0296G-AF488, 1:200); Goat Alexa Fluor 594 (Jackson, RRID: 115-585-003, 1:400); and Mouse Cy2 (CWBIO, CW0158, RRID: 1:400).

### Synaptoneurosomal Preparation

Syn-PER™ Synaptic Protein Extraction Reagents (Thermo Scientific, Waltham, USA, RRID: 87793) were used for synaptic protein extraction according to the manufacturer's standard protocol. Briefly, cultured neurons were washed twice with ice-cold 1XPBS, and 1 mL Syn-PER Reagent was added to a 100 mm cell plate. The plate surface was scraped using a cell scraper to lift the cells, and the lysate were centrifuged at 1,600 g for 10 min at 4°C, then the supernatant was transferred to a new tube. The supernatant was centrifuged at 15,000 g for 20 min at 4°C to obtain the synaptosome pellet. Finally, the synaptosome pellet was suspended in Syn-PER Reagent.

### Western Blot Analysis

Western blot analysis was conducted as previously reported (Tian et al., 2015). Briefly, protein concentrations were determined using BCA protein assay kit (Thermo Scientific, Waltham, USA). Equal amounts of total protein (10–20 μg/lane) were resolved on denaturing sodium dodecyl sulfate–polyacrylamide electrophoresis gels and transferred to polyvinylidene difluoride membranes (Bio-Rad) by electroblotting. The membranes were then blocked in 5% milk/PBS for 2 h at 25°C and incubated with primary antibodies overnight at 4°C in solution. After incubated for another 2 h with HRP-conjugated secondary antibodies, the immunoreactive bands were visualized with Clarity™ Western ECL Substrate (Bio-rad, USA, RRID: Cat# 1705060s). The signal value for the band of interest was normalized to that of glyceraldehyde-3-phosphate dehydrogenase (GAPDH) in the same lane. Primary antibodies used are as follows: Rabbit CLSTN1 (Santa, RRID: sc133315, 1:500); Goat ICAM5 (Santa, RRID: sc-22028, 1:500); Mouse PSD95 (Cell signaling Technology, RRID: 2507S, 1:1,000); Mouse actin (CWBIO, RRID: CW0095A, 1:1,000); and Mouse GAPDH (Proteintech, RRID: 6004-1-Ig, 1:10,000). Secondary antibodies used are as follows: Goat anti-rabbit IgG (Jackson, RRID: 115-035-003, 1:10,000); Goat anti-mouse IgG (Jackson, RRID: 111-035-003, 1:10,000); and Rabbit anti-goat IgG (Bioss, RRID: AEO12301, 1:1,000).

### Statistical Analysis

All data are presented as group means ± standard deviation (SD). For multi–group comparisons, one- or two-way analysis of variance (ANOVA) models, followed by Bonferroni *post-hoc* tests, were computed using Origin 7.5 (Origin Lab, Northampton, MA, USA). In cases where comparisons were between two groups, independent Student's *t*-tests were performed. All data analyses were performed blinded to the experimental condition. A two-sided *p*-value of < 0.05 indicated a statistically significant difference.

## Results

### ICAM5 and CLSTN1 Colocalize in Mouse Cortical Neurons

To investigate the mechanistic link between CLSTN1 and ICAM5, we conducted serial experiments *in vitro*. To begin with, we tested the cellular localization of CLSTN1 and ICAM5 protein in N2a cells. As shown in Figures S2A, S2B, both proteins were found in N2a cells with a similar size and distribution, revealing the co-localization. To further improve our understanding, we repeated it in primary cultured neurons. First, we double labeled 14 DIV neurons with microtubule associated protein 2 (MAP2, red) and fibrillary acidic protein (GFAP, green). Figure 1A shows that the majority of cells were neurons as they were labeled by MAP2 (red). Then, we stained cultured neurons using antibodies that specific against CLSTN1, ICAM5 and a disintegrin and metalloprotease 10 (ADAM10). ADAM10 was used as a negative control for CLSTN1 immunoisolates did not contain ADAM10 (Steuble et al., 2012). As expected, CLSTN1 colocalized with ICAM5 (pearson's correlation coefficient = 0.488), but not with ADAM10 (pearson's correlation coefficient = 0.112) (Figure 1B). We next quantified the proportion of CLSTN1 co-localized with ADAM10 or ICAM5 in the dendrites (19%, *df* = 11, *t* = −2.12; 60.84%, *df* = 11, *t* = −2.89, *p* < 0.05) (Figure 1C). These results support the observation that CLSTN1 clearly co-localized with ICAM5 in primary cultured neurons.

### EGFP-CLSTN1 and ICAM5-mCherry Co-transport in Mouse Cortical Neurons

**Figure 2:**
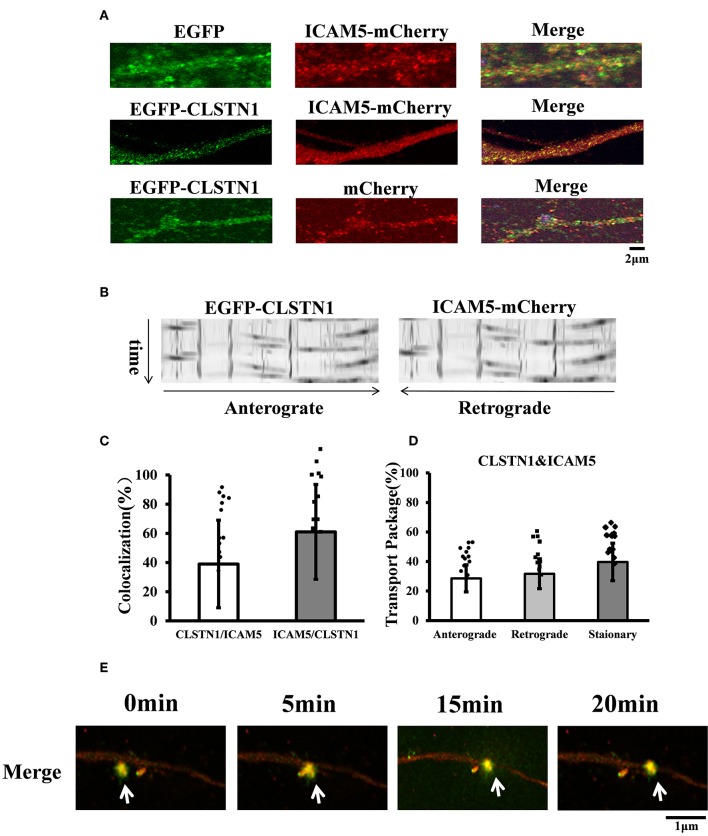
EGFP-CLSTN1 and ICAM5-mCherry co-localize and co-transport in dendrites. **(A)** Neurons were transfected with EGFP, mCherry, EGFP-CLSTN1 and ICAM5-mCherry at 3 DIV. Confocal images show the co-localization of EGFP-CLSTN1 and ICAM5-mCherry in dendritic areas of cultured neurons at 11 DIV. Scale bars = 2 μm, *n* = 12. **(B)** Representative kymographs of EGFP-CLSTN1- and ICAM5-mCherry- containing vesicles in the dendrites. Lentivirus of EGFP-CLSTN1 and ICAM5-mCherry were transfected into cultured neurons at DIV3. Live cells were imaged at 1 s/interval for 1 min using confocal microscopy. Kymographs of positive vesicles trafficking along dendrites were analyzed by ImageJ. Right or left descending particles represent anterograde or retrograde transport. **(C)** Quantification of co-localization was performed using the Image J software as described in the methods. Histogram represents the relative intensity of CLSTN1 and ICAM5. Data are shown as mean ± SD. **(D)** Transport of EGFP-CLSTN1- and ICAM5-mCherry- containing vesicles were quantified. Stationary, anterograde and retrograde vesicle transportation were quantified independently at each channel. In three independent experiments, a total of 201 EGFP-CLSTN1 and ICAM5-mCherry vesicles from 20 neurons were quantified. **(E)** Neurons were imaged every 5 min from 0 to 20 min using time-lapse imaging. White arrows show co-localization and co-transportation of EGFP-CLSTN1 and ICAM5-mCherry. Scale bars = 1 μm.

CLSTN1 protein has been proved to co-localize with amyloid precursor protein (APP) and to mediate transport of APP (Vagnoni et al., 2012). The co-localization of ICAM5 and CLSTN1 protein in both N2a cells and primary neurons suggests that two proteins would co-transport. Therefore, we monitored dendritic transport of ICAM5 and CLSTN1 protein using dual imaging time-lapse microscopy in cultured mouse neurons infected with lentivirus (ICAM5-mCherry and EGFP-CLSTN1) (*n* = 12). We cannot detect co-localization in neurons co-transfected EGFP with ICAM5-mCherry or mCherry with EGFP-CLSTN1, but high levels of co-localization of ICAM5 and CLSTN1 were detected in dendrites (Figure 2A). Furthermore, co-movement of ICAM5-mCherry and EGFP-CLSTN1 was observed through dendritic spines (Figures 2B–D). Kymographs showed about 62.6% (*df* = 11, *t* = −1.32) of EGFP-CLSTN1 pixels co-localized with ICAM5-mCherry pixels, and 39.7% (*df* = 11, *t* = −1.32) ICAM5 pixels co-localized and co-transported with CLSTN1 pixels in dendrites (Figures 2B,C). Approximately 28.6% (*df* = 11, *t* = −1.89) of ICAM5-Cherry and EGFP-CLSTN1 co-transported in the anterograde direction, approximately 31.7% (*df* = 11, *t* = −1.89) of ICAM5-Cherry and EGFP-CLSTN1 co-transported in the retrograde direction, and approximately 62.8% (*df* = 11, *t* = −1.67) of ICAM5-Cherry and EGFP-CLSTN1 moved non-significantly (Figure 2D). Figure 2E showed ICAM5-mCherry and EGFP-CLSTN1 moved quickly during the transportation (Figure 2E, arrows). These results indicate the co-transportation of CLSTN1 and ICAM5 in live neurons.

### shRNA-Mediated Downregulation of CLSTN1 Increases ICAM5 on the Surface of Dendrites and Changes ICAM5 Distribution

**Figure 3:**
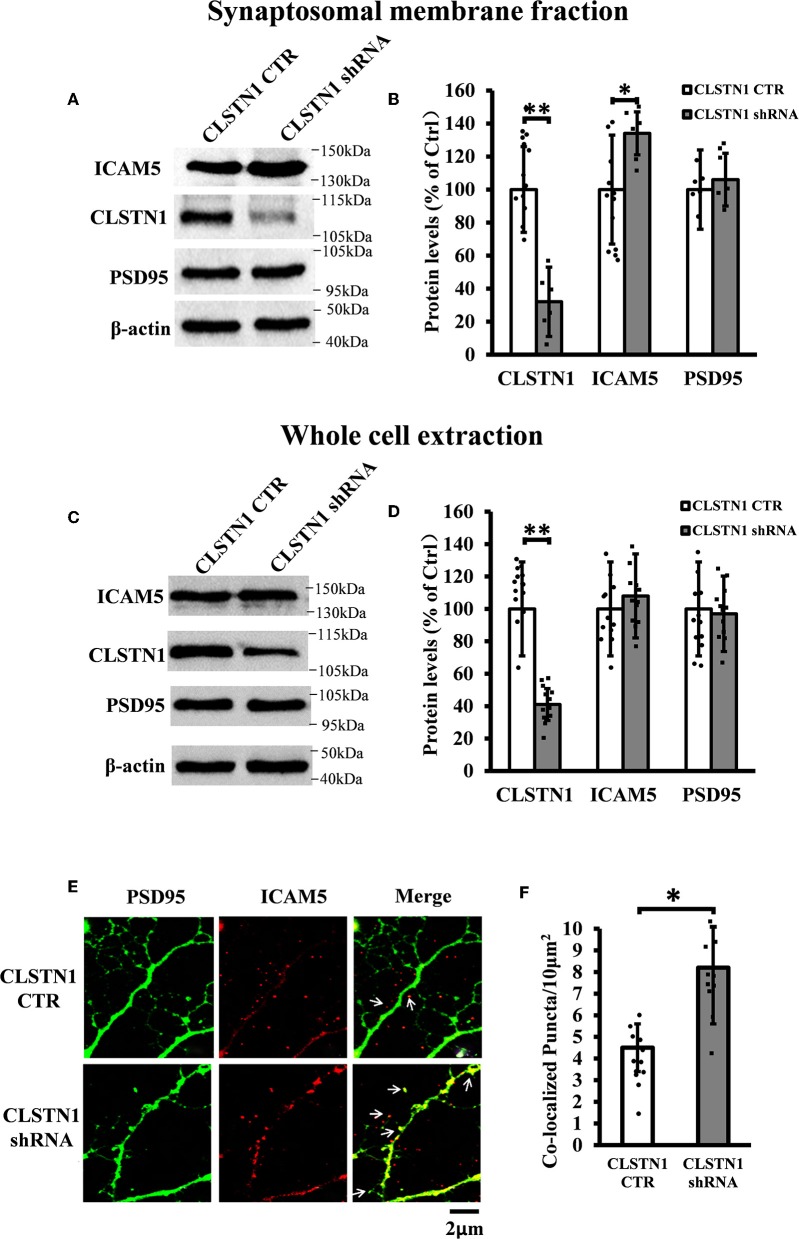
CLSTN1 shRNA increases ICAM5 expression on the surface of dendrites and changes ICAM5 distribution. **(A,C)** Representative western blot images for CLSTN1, ICAM5, and PSD95 from synaptosomal and whole cell extractions. PSD95 served as the postsynaptic marker. Neurons were transfected with empty plasmid (CLSTN1 CTR) or CLSTN1 shRNA constructs, *n* = 12. **(B,D)** Densitometric calculation and statistical analyses show that CLSTN1 shRNA downregulates the CLSTN1 protein and increases synaptosomal ICAM5. **(E)** Neurons were immunostained with ICAM5 (red) and postsynaptic marker PSD95 (green). White arrows show PSD95 and ICAM5 co-localized puncta. Scale bars = 2 μm. *n* = 12. **(F)** Quantification of PSD95 and ICAM5 co-localized puncta. CLSTN1 shRNA changes ICAM5 distribution. Data are shown as mean ± SD. *p < 0.05, **p < 0.01 compared to CLSTN1 CTR.

In order to reveal the function of CLSTN1 on ICAM5 activity and distribution, we downregulated CLSTN1 in cultured neurons from WT mice with the aid of recombinant lentiviral vectors expressing a specific short hairpin (sh)RNA. Crude subcellular fractions and whole cell extraction of cultured neurons were evaluated by western blot at 11 days post transfection (Figures 3A,C), and the postsynaptic density protein 95 (PSD95) served as a post-synapse marker. Under shRNA knockdown, we observed a significant decrease in CLSTN1 protein level by 67.53% (*df* = 5, *t* = −3.69, *p* < 0.01). Intriguingly, downregulation of CLSTN1 was associated with a significant increase of 34.64% (*df* = 6, *t* = −2.943, *p* < 0.05) in the level of synaptosomal ICAM5 (Figure 3B). Notably, the levels of whole cell ICAM5 remained largely unaffected (Figure 3D). These results may indicate that the loss of CLSTN1 results in excessive ICAM5 protein abundance on the surface of synaptosomal membrane fraction but not in the whole cell.

We next examined whether CLSTN1 shRNA can reduce ICAM5 processing and change the distribution of ICAM5 on dendrites by using immunocytochemistry. ICAM5 and PSD95 in cultured neurons were co-labeled 11 days post transfection (Figure 3E). CLSTN1 shRNA resulted in co-localization of ICAM5 and PSD95 on the surface of the postsynaptic membrane (Figure 3E). Knocking down CLSTN1 significantly increased the number of co-localized puncta of ICAM5 with PSD95 (34.2%, *df* = 11, *t* = −2.82, *p* < 0.05; pearson's correlation coefficient = 0.6432) (Figure 3F). Thus, it further validated the reduction of CLSTN1 could alter ICAM5 distribution patterns at postsynaptic membrane. This is the first observation that loss of CLSTN1 could alter the accumulation of ICAM5 at postsynaptic membrane.

### shRNA-Mediated Downregulation of CLSTN1 Affects Dendritic Morphology

**Figure 4:**
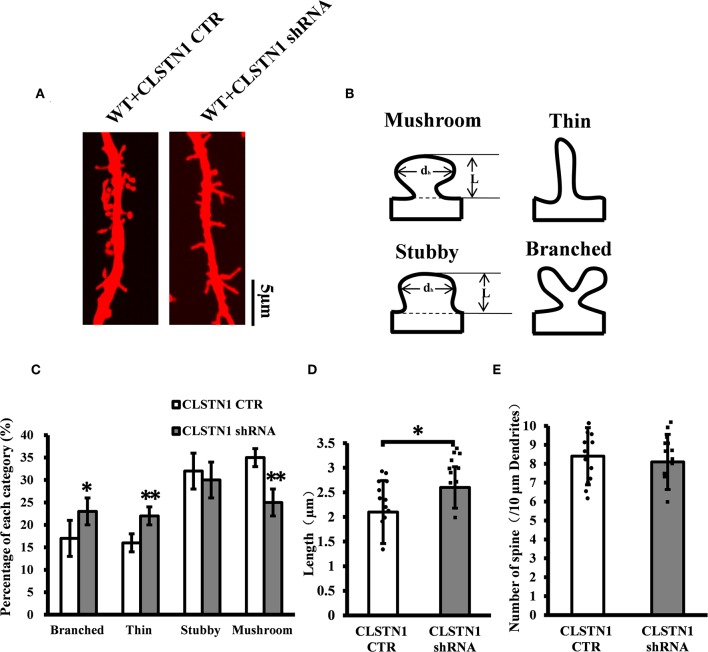
CLSTN1 shRNA affects dendritic morphology. **(A)** Representative images of Dil-stained neurons after transfection with CLSTN1 shRNA or CLSTN1 CTR. Each photograph represents a 20-μm-long dendritic segment, *n* = 12. **(B)** Categories of spine morphology, mushroom (spines with a large bulbous head; the spine head diameter is at least 1.5 μm and larger than the spine neck diameter); thin (filopodia-like protrusions; diameters of the spine and neck are nearly equal, and spine length is greater than spine width); stubby (short spines without a well-defined spine neck); branched (spines with more than one head). **(C–E)** Quantifications of dendritic morphology, spine length and spine number. CLSTN1 shRNA affects dendritic morphology. Data are shown as mean ± SD. *p < 0.05, **p < 0.01 compared to CLSTN1 CTR.

To verify the role of endogenous CLSTN1 proteins in dendrite morphogenesis, we transfected DIV 3 neurons with control vectors or CLSTN1 shRNA constructs, and analyzed neuronal morphology at 15 days after transfection. After quantifying dendritic spine number, spine length, and dendritic morphology, we found that downregulation of CLSTN1 appeared to impact spine maturation. More branched (28.1%, *df* = 191, *t* = 2.16, *p* < 0.05) and thin spines (32.1%, *df* = 191, *t* = −2.86, *p* < 0.01) and less mushroom spines (41.62%, *df* = 191, *t* = −3.16, *p* < 0.01) were found in CLSTN1 shRNA neurons (Figures 4A–C). CLSTN1 shRNA also affected the length of dendritic spines. The average length increased 13.3% in CLSTN1 shRNA neurons (13.3%, *df* = 191, *t* = −2.67, *p* < 0.05) (Figures 4A,D). However, CLSTN1 shRNA did not affect the number of dendritic protrusions (Figures 4A,E). These data indicate that disrupting the balance of CLSTN1 signaling can alter spine development in cultured cortical neurons.

### CLSTN1 Overexpression Decreases ICAM5 Expression in *Fmr1* KO Neurons

**Figure 5:**
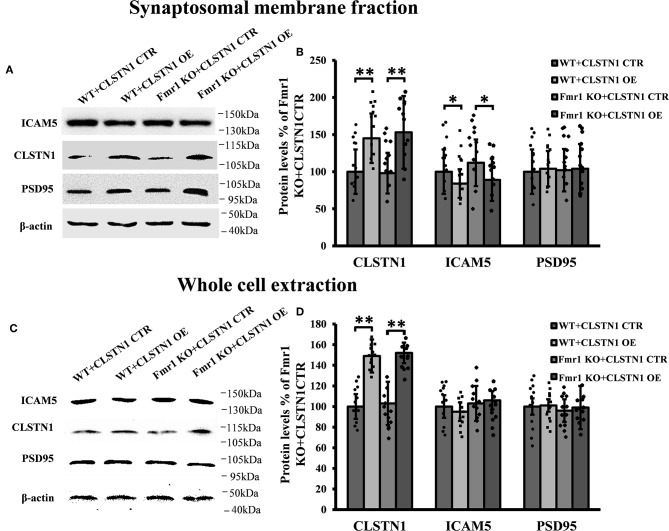
CLSTN1 overexpression normalizes ICAM5 expression in *Fmr1* KO neurons. **(A,C)** Representative western blot images for CLSTN1, ICAM5, and PSD95 expressions. Neurons were transfected with overexpression (CLSTN1 OE) or control plasmid constructs (CLSTN1 CTR), *n* = 12. **(B,D)** Significant increase of CLSTN1 was detected in neurons transfected with CLSTN1 OE, while ICAM5 was decreased in the synaptosomal membrane fraction transfected with CLSTN1 OE, But not in whole cell extraction.

Since shRNA-mediated downregulation of CLSTN1 affects dendritic spine maturation and increases ICAM5 on the surface of dendrites, we next examined whether overexpressing (OE) CLSTN1 can reduce ICAM5 expression in *Fmr1* KO neurons. Firstly, we determined that ICAM5 and CLSTN1 colocalized in *Fmr1* KO neurons (Figure S3). Then, we overexpressed CLSTN1 in cultured WT and *Fmr1* KO cortical neurons. Crude subcellular fractions of cultured neuron and whole cell extraction were evaluated by western blot at 11 days post-transfection (Figures 5A,C). CLSTN1 was abundant in the crude synaptosomal membrane fraction in both control (*n* = 6) and OE neurons (*n* = 6). CLSTN1 overexpression altered the levels of synaptosomal ICAM5 (Figure 5B) but not the levels of whole cell ICAM5 (Figure 5D) in cultured WT (17.2%, *df* = 5, *t* = 2.14, *p* < 0.05) and *Fmr1* KO neurons (22.4%, *df* = 5, *t* = 2.34, *p* < 0.05). These results indicate that the overexpression of CLSTN1 reduces the expression of ICAM5 in synaptosomal membrane fractions without influencing ICAM5 expression in the whole cells.

### CLSTN1 Overexpression Normalizes Dendritic Spine Morphology in *Fmr1* KO Neurons

**Figure 6:**
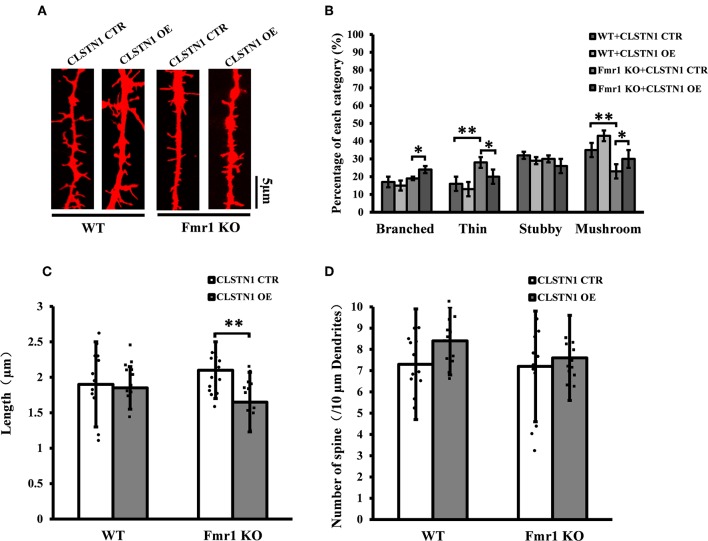
CLSTN1 overexpression rescues impaired dendritic spine phenotypes in *Fmr1* KO neurons. **(A)** Representative images of Dil-stained neurons transfected with CLSTN1 OE and CLSTN1 CTR in WT and *Fmr1* KO neurons. Each photograph represents a 20-μm-long spine segment. *n* = 12. **(B–D)** Quantifications of dendritic morphology, spine length and spine number. CLSTN1 overexpression rescues impaired dendritic spine phenotypes in *Fmr1* KO neurons. Data are shown as mean ± SD. *p < 0.05, **p < 0.01, compared to CLSTN1 CTR.

We further assessed the effect of overexpressing CLSTN1 on dendritic spine morphology in cultured WT and *Fmr1* KO neurons. Overexpressing CLSTN1 in neurons (*n* = 12) led to morphological maturation in dendritic spines (Figures 6A,B). Moreover, overexpressing CLSTN1 (*n* = 12) reduced thin spines (22.6%, *df* = 191, *t* = 2.02, *p* < 0.05) and increased mushroom spines (23.4%, *df* = 191, *t* = 2.02, *p* < 0.05) compared to control treatment in *Fmr1* KO neurons (*n* = 12) (Figure 6B). The decreased fraction of filopodia-like thin spines suggests that more immature structures were converted into mature spines and that spines were more stable in OE neurons. In addition, Overexpressing CLSTN1 also affected the length of dendritic spines in *Fmr1* KO neurons, with an average length decrease (19.4%, *df* = 191, *t* = −3.02, *p* < 0.01) (Figure 6C). Surprisingly, CLSTN1 overexpression did not alter spine number (Figure 6D), which suggests that CLSTN1 is not involved in the formation of nascent protrusions in *Fmr1* KO neurons. These results indicate overexpressing CLSTN1 can rescue spine immaturation in *Fmr1* KO neurons.

### The Expression of CLSTN1 and ICAM5, and Dendritic Spine Morphology in the Postnatal Medial Prefrontal Cortex of *Fmr1* KO Mice

**Figure 7:**
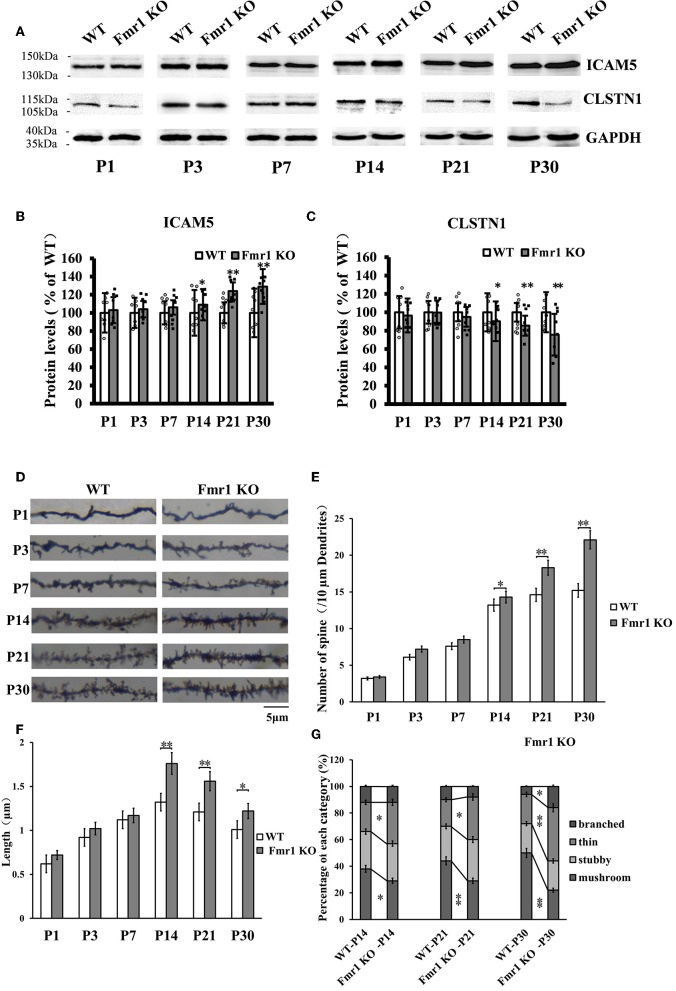
Clstn1 expression decreases in the postnatal medial prefrontal cortex of *Fmr1* KO mice while ICAM5 is highly expressed. **(A)** Representative western blot images for CLSTN1 and ICAM5 proteins from the medial prefrontal cortex of WT and *Fmr1* KO mice at P1 to P30 (*n* = 8 animals each time point × 6 time points). GAPDH in lower panel served as loading control. **(B,C)** Densitometric calculation and statistical analyses were performed. The reduction of CLSTN1 protein in the postnatal medial prefrontal cortex of *Fmr1* KO mice is associated with increased expression of ICAM5. Data are shown as mean ± SD, *p < 0.05, **p < 0.01 compared to WT group. **(D)** Representative images of Golgi-stained neurons in medial prefrontal cortex of *Fmr1* KO and WT control mice (*n* = 192 neurons each time point × 6 time points) between postnatal day 1 (P1) and postnatal day 30 (P30). Each photograph represents a 25-μm-long dendritic segment. **(E)** Spine number in pyramidal neurons of the medial prefrontal cortex was measured. **(F)** Spine length in pyramidal neurons of the medial prefrontal cortex medial was measured. **(G)** The proportion of different morphology was calculated using the following categories: branched (spines with more than one head), thin (filopodia-like protrusions), stubby (short spines without a well-defined spine neck), and mushroom (spines with a large bulbous head). Data are shown as mean ± standard error of mean (SEM). *p < 0.05, **p < 0.01 compared to WT group. Scale bar = 5 μm. *n* = 4/group.

The above studies, which show that CLSTN1 negatively regulates ICAM5 expression and influences dendritic spine morphology in WT and *Fmr1* KO neurons, suggest the mechanistic link between CLSTN1 and ICAM5 implicated in FXS. To further investigate how both proteins are altered in FXS within postnatal brain development, we measured their expression at protein level in medial prefrontal cortex (mPFC) which is the critical brain region that involved in FXS. To obtain a temporal expression profiling, we detected the CLSTN1 and ICAM5 proteins expression in the mPFC of *Fmr1* KO mice from P1 to P30, and WT at the same age served as control (*n* = 8/group animals for each time point). As the results, we observed no difference in ICAM5 protein expression between WT and *Fmr1* KO mice at P1, P3, and P7. Whereas, there is a significant increase of ICAM5 in the *Fmr1* KO mice starting from P14 (16.84%, *df* = 7, *t* = −1.89, *p* < 0.05), and continues at P21 (20.2%, *df* = 7, *t* = −2.98, *p* < 0.01) and P30 (30.55%, *df* = 7, *t* = −3.12, *p* < 0.01) (Figures 7A,B). On the other hand, CLSTN1 protein shows a distinct decreasing expression profile, which is correlated to ICAM5 increase, in the *Fmr1* KO mice at P14 (16.90%, *df* = 7, *t* = −2.32, *p* < 0.05), P21 (19.79%, *t* = −2.98, *df* = 7, *p* < 0.01) and P30 (24.55%, *df* = 7, *t* = −3.12, *p* < 0.01) (Figures 7A,C). We also observed CLSTN1 and ICAM5 mRNA level in WT and *Fmr1* KO mPFC using qRT-PCR (Figure S4). However, there were no significant differences in transcript level between WT and *Fmr1* KO mice, suggesting that FMRP regulates CLSTN1 and ICAM5 mRNA translation, but not transcription. Collectively, CLSTN1 protein level is declined in the mPFC of *Fmr1* KO mice while ICAM5 is upregulated during postnatal development, which matches the impairments of dendritic spine maturation (Figures 7D–G) from P14 in *Fmr1* KO mice. As shown in Figures 7D,E, both *Fmr1* KO and WT mice showed an increase in the number of dendritic spines in the cortex from P1 to P30. The number was higher in KO mice at the following points: P14 (18.89%, *df* = 47, *t* = −1.78, *p* < 0.05), P21 (26.22%, *df* = 47, *t* = −3.28, *p* < 0.01), and P30 (31.22%, *df* = 47, *t* = −3.14, *p* < 0.01). The length of dendritic spines in the cortical neurons increased from P1 to P14 and then decreased from P14 to P30. However, KO mice showed relatively longer spines than WT mice at the following points: P14 (34.2%, *df* = 47, *t* = −1.44, *p* < 0.05), P21 (24.56%, *df* = 47, *t* = −1.94, *p* < 0.05), and P30 (21.61%, *df* = 47, *t* = −2.93, *p* < 0.01) (Figure 7F). We further examined the spine morphology using the following categories: branched (spines with more than one head), thin (filopodia-like protrusions), stubby (short spines without a well-defined spine neck), and mushroom (spines with a large bulbous head) (Figure 7G). The results showed that KO mice exhibited an increase in the number of thin-headed spines in the medial prefrontal cortex compared with WT at P14 (29.03%, *df* = 47, *t* = −1.53, *p* < 0.05), P21 (37.50%, *df* = 47, *t* = −1.46, *p* < 0.05), and P30 (45.00%, *df* = 47, *t* = −3.46, *p* < 0.01) and a decrease in the number of mushroom spines at P14 (31.03%, *df* = 47, *t* = −2.26, *p* < 0.05), P21 (51.72%, *df* = 47, *t* = −3.26, *p* < 0.01), and P30 (89.56%, *df* = 47, *t* = −3.45, *p* < 0.01), suggesting a delay in filopodia-to-spine transition and a deficit in spine maturation. Similarly, in hippocampus CLSTN1 expression decreases while ICAM5 is highly expressed in the postnatal *Fmr1* KO mice, and we can observe the number of immature dendritic spine was higher in *Fmr1* KO mice started from P14 to P30 (Figure S5). In all, these data suggest the alteration in CLSTN1 and ICAM5 may correlate with the excessive filopodia-like spines in *Fmr1* KO mice.

## Discussion

Both CLSTN1 and ICAM5 were previously identified by HITS-CLIP as FMRP targets (Darnell et al., 2011), but subsequent analyses of their molecular mechanisms linked to FXS or FMRP are lacking. In this study, using both *in vivo* and *in vitro* approaches, we demonstrated a key role of CLSTN1 in mediating the intracellular redistribution of ICAM5 within neurons, in which, it subsequently affects the maturation of dendritic spines in the normal brain. Whereas, CLSTN1 dysregulation contributes to excessive dendritic ICAM5 distribution and promotes abnormal spine formation in *Fmr1* KO mice. As a negative regulator of filopodia to spine transition (Matsuno et al., 2006) by facilitating filopodia formation and slowing down spine maturation (Furutani et al., 2011), ICAM5 is highly expressed in dendritic filopodia. Previous studies have suggested that CLSTN1, similar to other synaptic adhesion molecules, plays a role in stabilizing the connection between dendritic filopodia and boutons (Steuble et al., 2012; Bian et al., 2015) and is involved in synapse formation as well as synaptic plasticity (Hoerndli et al., 2009; Vagnoni et al., 2012), which is characterized as a positive regulator in dendritic filopodia. We hypothesized that the interaction between CLSTN1 and ICAM5 were involved in the regulation of dendritic spine maturation. To test this hypothesis, we investigated the functional link between CLSTN1 and ICAM5 by conducting *in vitro* experiments. First, we analyzed the distribution of endogenous CLSTN1 and ICAM5 in 18 DIV cortical neurons and found that they have closely overlapped distribution along the dendritic shaft and within dendrites. This finding is further supported by the observation that CLSTN1 and ICAM5 have a more homogeneous surface distribution in N2a cells.

Previous studies reported that CLSTN1 acts as a ligand to mediate transport of vesicles by linking to Kinesin-1 motors, the known motor proteins (Konecna et al., 2006; Ster et al., 2014). Moreover, CLSTN1 have been proved to co-localize with amyloid precursor protein (APP) in cells and tissues, and CLSTN1 mediated axonal transport of APP (Ster et al., 2014), Rab5-containing endosomes, and NMDA receptors (Um et al., 2014). To investigate the function of localization of CLSTN1 and ICAM5, and the possible co-transport, we conducted live cell imaging studies. The results suggest that a significant proportion of ICAM5 is co-transported with CLSTN1 through dendrites, indicating that CLSTN1 is functionally involved in intracellular transport of ICAM5 *in vitro*. However, the precise mechanisms need further research.

CLSTN1 downregulation resulted in increased expression of ICAM5 protein on the surface of neurons, enhanced co-localization of ICAM5 with PSD95, and increased the presence of co-located points on the surface of dendritic spines. Thus, the reduction of CLSTN1 in cortical neurons changed the distribution pattern of ICAM5 and increased ICAM5 accumulation on postsynaptic terminals. This alteration in the spatial distribution of ICAM5 might be due to a disruption in CLSTN1-mediated dendritic transport of ICAM5. In cultured WT neurons, CLSTN1 downregulation led to a significantly increased proportion of stubby spines and a reduced proportion of mature dendritic spines. Collectively, these findings indicate that CLSTN1 mediates the transport of dendritic ICAM5-containing organelles in cultured neurons and further regulates dendritic spine maturation. In the absence of CLSTN1, the transport of ICAM5 is compromised, leading to dendritic spine abnormality.

When CLSTN1 protein expression was increased in cultured *Fmr1* KO neurons, spine maturation were improved, which suggests that filopodia, the precursors of spines (Chetkovich et al., 2002), transited to steady-state spines. We did not observe an increase in the total number of all type spines, which suggests that overexpressing CLSTN1 improved spine maturation in *Fmr1* KO neurons but was not involved in the formation of nascent protrusions in *Fmr1* KO neurons. Overexpressing CLSTN1 also led to the reduction of synaptosomal ICAM5 protein. Taken together, these findings suggest that CLSTN1 is significantly related to the functional activity of ICAM5, which in turn impacts the maturation of dendritic spines. However, the effects of overexpression of CLSTN1 with viral vectors *in vivo* on the dendritic spines, synaptic plasticity and behaviors in *Fmr1* KO mice need to research deeply.

Furthermore, we studied whether ICAM5 and CLSTN1 expression are changed during brain development *in vivo* in a mouse model of FXS, and whether such change is associated with altered spine morphology. We detected ICAM5 protein in the mPFC of *Fmr1* KO mice at different developmental stages (P1–P30) and found that it is excessively expressed. However, CLSTN1 protein levels are downregulated in the postnatal developmental KO mice. Previous studies showed that protein level changes identified by western blot or global proteomic approaches in FXS models, and some conflicting results have been shown which might be explained by the age or tissue examined etc. (Schutt et al., 2009; Darnell and Klann, 2013). Most FMRP target proteins were increased in its absence, however, recent examples of apparently decreased levels of FMRP targets have been observed, such as NR1, NR2A, NR2B, and SAPAP355 (Krueger et al., 2011; Till et al., 2012). In our study, the precise molecular mechanisms underlying CLSTN1 protein levels in the absence of FMRP require further investigation. *Fmr1* KO mice show a lower ratio of CLSTN1/ICAM5 expression in the medial prefrontal cortex at P21 and P30, when there was an increase in the proportions of immature filopodia-like dendritic protrusions in the neurons. Our findings are consistent with this and suggest ICAM5 and CLSTN1 are implicated in dendritic spine abnormalities in *Fmr1* KO mice.

In summary, this study provides the first evidence that CLSTN1 plays a critical role in the development of spines in *Fmr1* KO mice by modulating dendritic ICAM5 distribution, which is essential for dendritic spine maturation and function. Since both CLSTN1 and ICAM5 are FMRP targets, these results shed light on the mechanistic function of CLSTN1 and ICAM5 in dendritic spine genesis in an animal model of FXS. We propose that reduced CLSTN1 or excessive ICAM5 during brain development leads to aberrations in dendritic spine formation in FXS, which may contribute to neuropathology phenotypes associated with synaptic abnormality, such as learning disabilities. Future work may investigate the reason for aberrant alteration in CLSTN1 in FXS, determine the factors that regulate CLSTN1 to initiate ICAM5 transportation, and provide mechanistic insights into how dysfunctional CLSTN1 signaling pathway contributes to neurodevelopmental disorders.

## Data Availability Statement

The datasets generated for this study are available on request to the corresponding author.

## Ethics Statement

This study was carried out in accordance with the recommendations of National Institutes of Health (NIH) Guide for the Care and Use of Laboratory Animals, and Wuhan University of Science & Technology ethics committee with the number IACUC-2017032. The protocol was approved by Wuhan University of Science & Technology ethics committee.

## Author Contributions

KC, YC, and YZ conceived and designed the experiments. KC, CY, SZ, Y-PP, and DL performed the experiments. GC and LX provide the reagents and materials. YC, HD, and YZ wrote and revised the manuscript.

### Conflict of Interest

The authors declare that the research was conducted in the absence of any commercial or financial relationships that could be construed as a potential conflict of interest.

## Abbreviations

CLSTN1: Calsyntenin-1
ICAM5: Intercellular adhesion molecule 5
FXS: Fragile X Syndrome
MMP-2: matrix metalloprotein-2
APP: amyloid precursor protein
ADAM10: a disintegrin and metalloprotease 10
NMDA: N-methyl-D-aspartic acid receptor
ECL: chemiluminescence
Dil: 1,1′-dioctadecyl-3,3,3′,3′-tetramethylindocarbocyanine perchlorate
BSA: bovine serum albumin BSA
DAPI: 4′-6-Diamidino-2-phenylindole
GAPDH: glyceraldehyde-3-phosphate dehydrogenase
EGFP: Enhanced Green Fluorescent Protein
KLC: kinesin light chain-1
OE: overexpressing
N2a: Neuro-2a
DIV: days *in vitro*
KO: *Fmr1* knock out
WT: wild-type
MAP2: microtubule associated protein 2
GFAP: glial fibrillary acidic protein
HITS-CLIP: high throughput sequencing of RNAs isolated by crosslinking immunoprecipitation.
