# Supplementary material for: Calsyntenin-1 Negatively Regulates ICAM5 Accumulation in Postsynaptic Membrane and Influences Dendritic Spine Maturation in a Mouse Model of Fragile X Syndrome
Source: Front Neurosci. 2019 Oct 18;13:1098. doi: 10.3389/fnins.2019.01098 (PMC6813673; doi:10.3389/fnins.2019.01098)
Supplement: Supplementary file 1 [file Data_Sheet_1.pdf]

Supplemental results

Interference efficiency of siCLSTN1 in Neuro-2a (N2a) cells

We first confirmed the efficiency of three siRNA in reducing endogenous CLSTN1 expression in N2a cells transfected with CLSTN1 siRNA or negative control siRNA. The expression of CLSTN1 was markedly reduced by three CLSTN1 siRNA (Fig. S1). We chose siClstn1-3 for the next experiment.

Co-localization of ICAM5 and CLSTN1 in N2a cells

We observed the co-localization of ICAM5 and CLSTN1 when co-expressing the two proteins in Neuro-2a (N2a) cell lines (Figure S2A and S2B) (n = 8). Consistently, both proteins were found in N2a cells with a similar size and distribution, and they revealed a high degree of co-localization. This result supports the observation that CLSTN1 and ICAM5 have a significant homogeneous surface distribution in primary cultured neurons.

Co-localization of ICAM5 and CLSTN1 in *Fmr1* KO neurons

we applied immunocytochemistry staining with antibodies that are specific against CLSTN1 and ICAM5 to detect them in *Fmr1* KO neurons. Figure S3A showed that both CLSTN1 and ICAM5 were enriched in *Fmr1* KO neuronal dendrites (Figure S3A). We then quantified the proportion of CLSTN1 co-localized with ICAM5 in the

dendrites. CLSTN1 clearly co-localized with ICAM5 (Figure S3B).

The mRNA levels of ICAM5 and CLSTN1 in the postnatal medial prefrontal cortex of WT and *Fmr1* KO mice

To determine the transcript level of ICAM5 and CLSTN1 in the postnatal medial prefrontal cortex, we examined mRNA expression of *Fmr1* KO mice and WT from P1 to P30 (n = 6/group). Figure S4A and S4B shows the mRNA expressions of ICAM5 and CLSTN1 were unchanged in the medial prefrontal cortex between WT and *Fmr1* KO mice from P1 to P30 (Figure S4A and S4B).

The expression of CLSTN1 and ICAM5, and dendrite and spine morphology in the postnatal hippocampus of Fmr1 KO mice

As shown in Fig.S5A and S5B, we observed no difference in ICAM5 protein expression between WT and *Fmr1* KO mice at P1, P3, P7 and P14. Whereas, there was a significant increase of ICAM5 in the *Fmr1* KO mice at P21 (34.84%, df = 7, t = -1.42, $p < 0.05$), and P30 (40.2%, df = 7, t = -2.13, $p < 0.01$) (Figure S5A and S5B). CLSTN1 protein remained a significant decreasing expression profile in the *Fmr1* KO mice at P7 (22.90%, df = 7, t = -2.32, $p < 0.05$), P14 (18.45%, df = 7, t = -2.32, $p < 0.05$), P21 (29.33%, t = -2.98, df = 7, $p < 0.05$) and P30 (70.33%, df = 7, t = -3.32, $p < 0.01$) (Figure S5A and S5C). To determine the age-dependent morphological changes in spines in *Fmr1* KO mice, we examined dendritic spines on Golgi-impregnated pyramidal type neurons at hippocampus of *Fmr1* KO mice and WT controls (n =175 neurons for each time point). Figure S5D shows the representative dendrite images of Golgi-stained pyramidal neurons from hippocampal P1 to P30. As shown in Figure

S5E, both *Fmr1* KO showed an increase compared with WT mice in the number of dendritic spines at P14 (48%, df = 47, t = -1.96, P < 0.01), P21 (29.22%, df = 47, t = -2.18, P < 0.01), and P30 (20.22%, df = 47, t = -3.14, P < 0.01). The length of dendritic spines in hippocampus showed longer spines than WT mice at P14 (24.2%, df=47, t = -1.44, P < 0.01), P21 (34.46%, df=47, t = -1.92, P < 0.01), and P30 (38.62%, df=47, t = -2.91, P < 0.01) (Figure S5F).

We further examined the spine morphology and the results showed that KO mice exhibited an increase in the number of thin-headed spines in the hippocampus compared with WT at P21 (22.50%, df=47, t = -1.42, P < 0.01), and P30 (22.00%, df=47, t = -3.36, P < 0.01) and a decrease in the number of mushroom spines at P21 (31.72%, df=47, t = -3.26, P < 0.01), and P30 (69.56%, df=47, t = -3.45, P < 0.01), suggesting a delay in filopodia-to-spine transition and a deficit in spine maturation.

Figure. S1

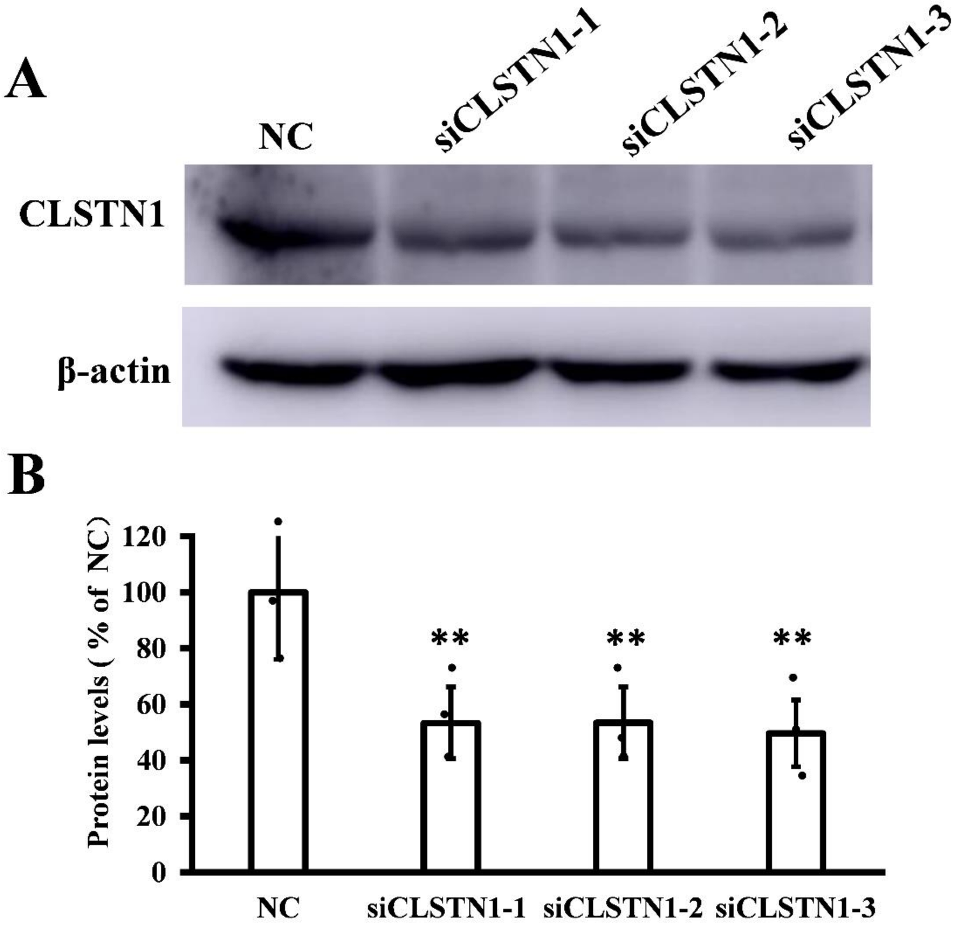
A
NC
siCLSTN1-1
siCLSTN1-2
siCLSTN1-3
CLSTN1
β-actin
B
Protein levels (% of NC)
120
100
80
60
40
20
0
**
**
**
NC
siCLSTN1-1
siCLSTN1-2
siCLSTN1-3

Figure. S2

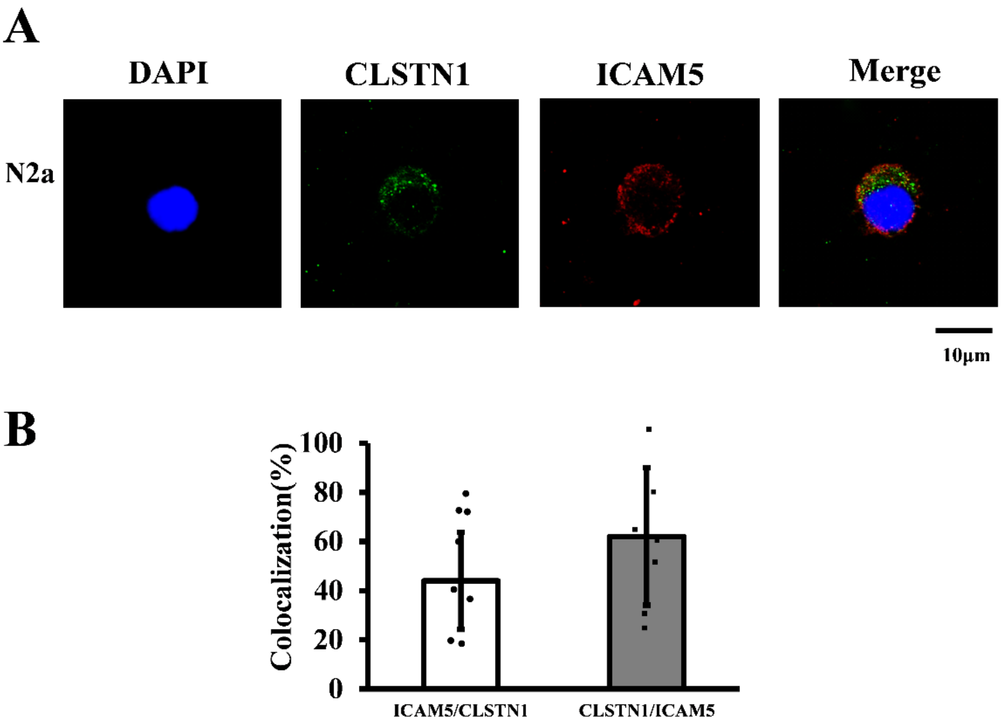
A
DAPI
CLSTN1
ICAM5
Merge
N2a
10μm
B
Colocalization(%)
100
80
60
40
20
0
ICAM5/CLSTN1
CLSTN1/ICAM5

Figure. S3

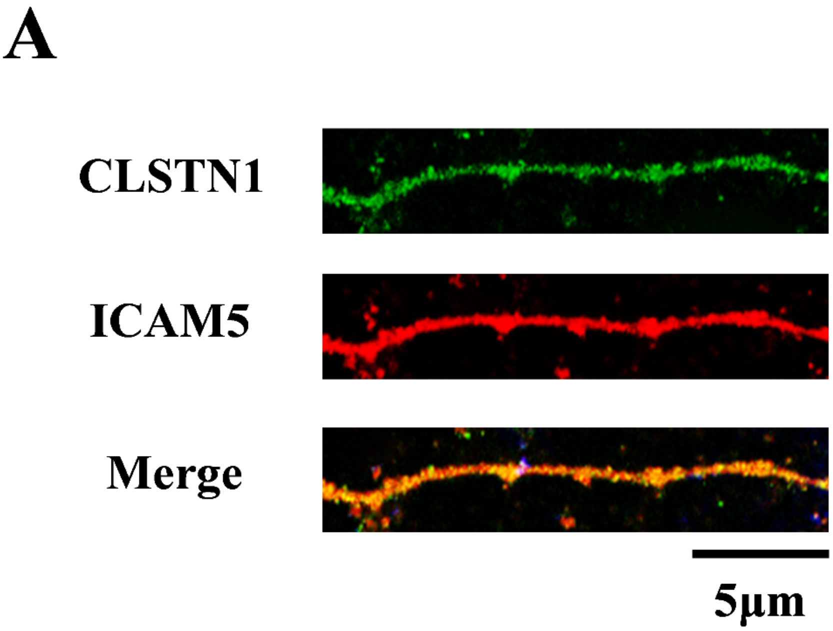
A
CLSTN1
ICAM5
Merge
5μm

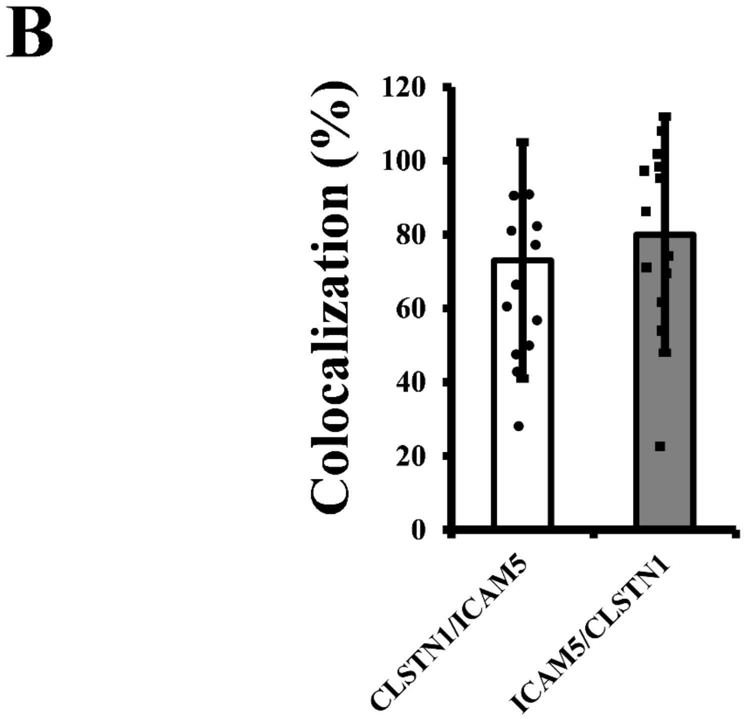
B
Colocalization (%)
120
100
80
60
40
20
0
CLSTN1/ICAM5
ICAM5/CLSTN1

Figure. S4

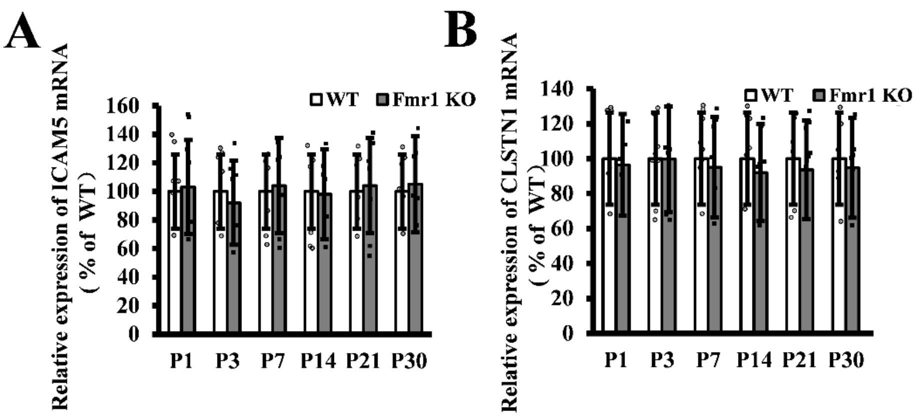
A
Relative expression of ICAM5 mRNA
(% of WT)
WT
Fmr1 KO
160
140
120
100
80
60
40
20
0
P1 P3 P7 P14 P21 P30
B
Relative expression of CLSTN1 mRNA
(% of WT)
WT
Fmr1 KO
140
120
100
80
60
40
20
0
P1 P3 P7 P14 P21 P30

Figure. S5

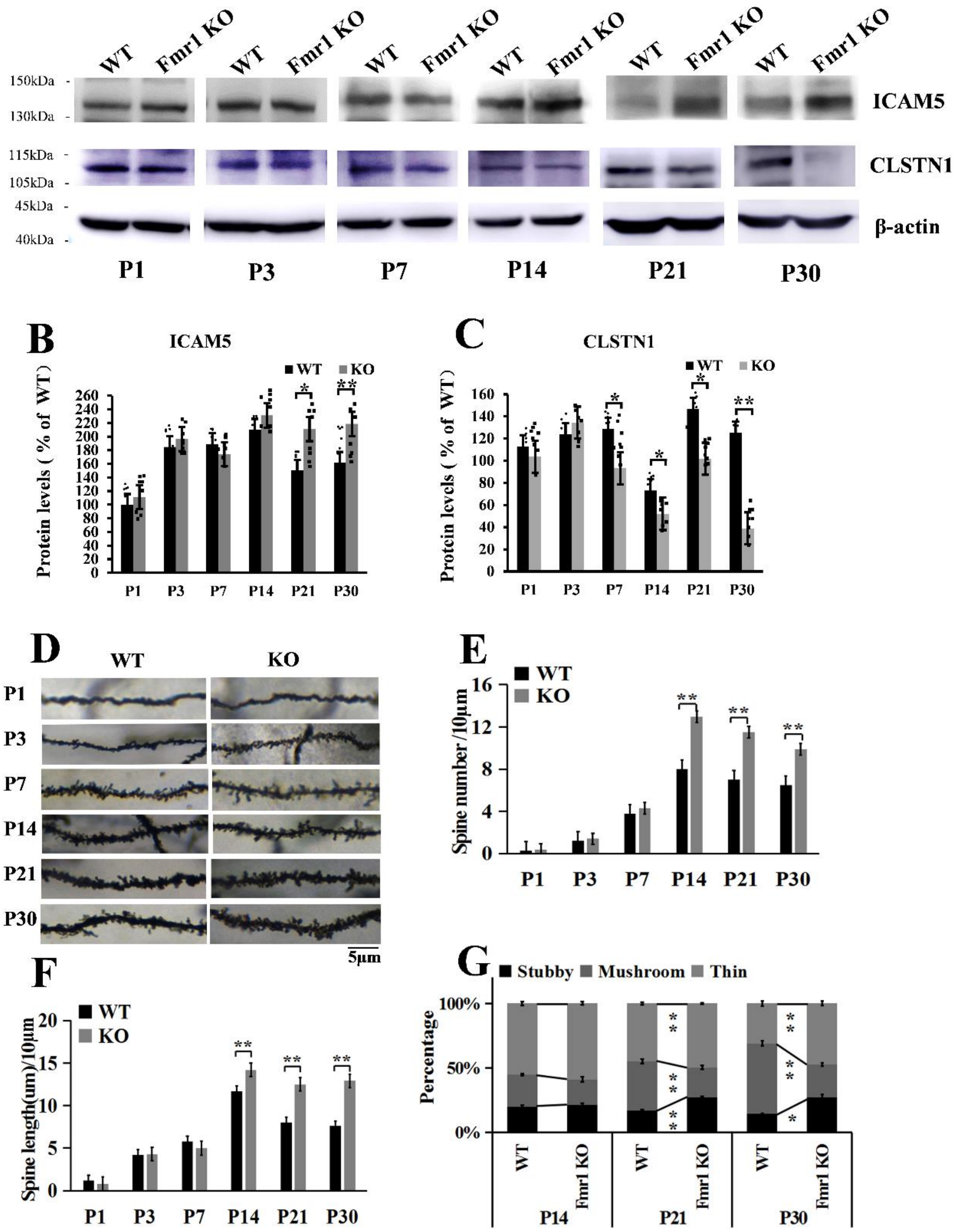

WT
Fmr1 KO
150kDa
130kDa
115kDa
105kDa
45kDa
40kDa
ICAM5
CLSTN1
β-actin
P1
P3
P7
P14
P21
P30
B
ICAM5
WT
KO
Protein levels (% of WT)
C
CLSTN1
Protein levels (% of WT)
D
WT
KO
5μm
E
Spine number /10μm
F
Spine length(um)/10μm
G
Stubby
Mushroom
Thin
Percentage
100%
50%
0%

Supplemental figure legends

Figure S1. Interference efficiency of siCLSTN1 in Neuro-2a (N2a) cells. **(A)** N2a cells were transfected with siCLSTN1-1, siCLSTN1-2 and siCLSTN1-3 or negative control (NC). After 36h, whole cell extractions were prepared and subjected to Western blot analysis with antibodies specific for CLSTN1. Anti-β-actin was included as a control for sample loading. **(B)** A bar graph presenting grey density values showing CLSTN1 expression. $^{**}p < 0.01$ compared to NC group. n =3.

Figure S2. Co-localization of CLSTN1 and ICAM5 in N2a cells. **(A)** N2a cells were immunostained for CLSTN1 (green) and ICAM5 (red). Confocal images show the co-localization (white). Scale bars = 10 μm. **(B)** Quantification of co-localization was performed using the Image J software. Histograms represent the relative intensity of CLSTN1 and ICAM5. Data are shown as mean ± SEM. n = 8/group.

Figure S3. CLSTN1 co-localizes with ICAM5 in *Fmr1* KO neurons. **(A)** Co-localization of CLSTN1 and ICAM5 is detected in dendrites (n = 12). Scale bars = 2 μm. **(B)** Quantification of co-localization was performed in dendrites using the Image J software as described in the methods. Histograms represent the relative intensity of CLSTN1 and ICAM5. Data are shown as mean ± SD.

Figure S4. qRT-PCR analysis of ICAM5 **(A)** and CLSTN1 **(B)** mRNA in the prefrontal cortex of WT and *Fmr1* KO mice from P1 to P30 (n=6/group). The data were presented as mean ± SEM from three independent experiments.

Figure S5. The expression of ICAM5 and CLSTN1 and dendritic morphology in the developmental hippocampus of WT and *Fmr1* KO mice. **(A)** Representative western blot images for CLSTN1 and ICAM5 proteins from the hippocampus of WT and *Fmr1* KO mice at P1 to P30 (n = 8 animals each time point × 6 time points). β-actin served as loading control. **(B and C)** Graph showing the densitometric quantification of CLSTN1 and ICAM5 protein. Data are shown as mean ± SD, $^{*}p<0.05$, $^{**}p < 0.01$ compared to WT group. **(D)** Representative images of Golgi-stained neurons in hippocampus of *Fmr1* KO and WT control mice (n= 175 neurons each time point × 6 time points) between postnatal day 1 (P1) and postnatal day 30 (P30). Each photograph represents a 25-μm-long dendritic segment. **(E)** Spine number in pyramidal neurons of the hippocampus was measured. **(F)** Spine length in pyramidal neurons of the hippocampus was measured. **(G)** The proportion of different morphology was calculated using the following categories: branched (spines with more than one head), thin (filopodia-like protrusions), stubby (short spines without a well-defined spine neck), and mushroom (spines with a large bulbous head). Data are shown as mean ± standard error of mean (SEM). *p < 0.05, **p < 0.01 compared to WT group. Scale bar = 5μm. n =4/group.

Supplementary table.

Table S1. siRNA sequence and target sequence of CLSTN1.

siRNA name	siRNA sequence	Target sequence
siClstn1-1	5’- UUCAUAUCUGUCUUAUCCGAA -3’	5’- TTCGGATAAGACAGATATGAACC -3’
siClstn1-2	5’- UAGUCUUUCUGUAGUUCACAG -3’	5’- CTGTGAACTACAGAAAGACTACA -3’
siClstn1-3	5’- UUGACGCUUAUCUUCACUA -3’	5’- TAGTGAAGATAAGCGTCAA -3’

Table S2. Forward/reverse primers used for qRT-PCR.

Gene name	Forward (5’-3’)	Reverse (5’-3’)
β-actin	5’-CTCTTTTCCAGCCTTCCTTCTTG-3’	5’-AGAGGTCTTTACGGATGTCAACG-3’
ICAM5	5’-GATCCTAGGCATCTCAGCG-3’	5’-GACAGTTAGTGCTGCAGTTGAG-3’
CLSTN1	5’-TAGTCACCGAGAACGATAACAC-3’	5’-TTTTGACCGGATTATTCCCTCA-3’
